# Long-Term Promotive and Protective Effects of Early Childcare Quality on the Social–Emotional Development in Children

**DOI:** 10.3389/fpsyg.2022.854756

**Published:** 2022-05-09

**Authors:** Corina Wustmann Seiler, Fabio Sticca, Olivia Gasser-Haas, Heidi Simoni

**Affiliations:** ^1^Research Department, Marie Meierhofer Children’s Institute (MMI), Zurich, Switzerland; ^2^Department of Pre-Primary and Lower Primary Level, Zurich University of Teacher Education (PH Zurich), Zurich, Switzerland; ^3^Institute for Educational Support for Behaviour, Socio-Emotional, and Psychomotor Development, University of Teacher Education in Special Needs (HfH), Zurich, Switzerland

**Keywords:** childcare quality, family risks, social–emotional development, preschool children, childcare centers, longitudinal, protective, promotive

## Abstract

The present study aimed to examine the longitudinal promotive and protective role of process quality in regular early childhood education and care (ECEC) centers in the context of early cumulative family risks on children’s social–emotional development from early to middle childhood. The sample consisted of 293 (T1; *M*_age_ = 2.81), 239 (T2; *M*_age_ = 3.76), and 189 (T3; *M*_age_ = 9.69) children from 25 childcare centers in Switzerland. Fourteen familial risk factors were subsumed to a family risk score at T1. Parents and teachers reported on children’s conduct problems (CP), emotional problems (EP), and prosocial behavior (PB) at T2 and T3. Childcare process quality was assessed at T2 using external observations of teaching and interaction, provisions for learning, and key professional tasks. Results showed that early family risks were positively associated with CP and EP and negatively associated with PB in the long term. High-quality teaching and interaction as well as caregivers’ professional behavior in terms of systematic observation, documentation, and planning of children’s individual learning processes and needs protected children from the undesirable long-term effects of early family risks on conduct problems, emotional problems, and prosocial behavior from early to middle childhood. The results indicate that a high process quality in ECEC might serve as an essential contextual protective factor in the development of resilience in children at risk.

## Introduction

Multiple early family adversities are a significant risk factor for child development (Rutter, 1990). However, the construct of resilience has decisively changed our notions of a positive development despite those risks. It describes, considering ecological systems theory of Bronfenbrenner (1979), a complex interplay of individual and contextual factors that support children’s adaptive development in the face of adverse experiences in the family (Luthar, 2015; Masten, 2018). Resilience refers both to the quality of the interaction between the child and his or her environment and to the competence of each side to provide what is needed for sustaining well-being and positive development (Ungar et al., 2013). An appropriately resourced environment in the micro- and mesosystem makes it more likely that the child’s motivation, disposition, and special talents will contribute to successful developmental outcomes. In this context, the role of significant other caregivers (e.g., childcare professionals or teachers) and stimulating experiences outside the family, such as in early childhood education and care (ECEC) settings, are seen as central contextual components of the dynamic resilience system (Werner and Smith, 2001). Because most children now attend regular center-based ECEC in the first years of their life (e.g., in a childcare center, playgroup, or nursery), ECEC could be considered a public health intervention that can benefit families, especially high-risk families, in the long term (Mortensen and Barnett, 2016). There is a body of evidence that extrafamilial social contexts can compensate for less stimulation at home, but in many cases based on highly specialized intervention programs (e.g., Brooks-Gunn, 2003; Nelson et al., 2003; Watamura et al., 2011; Melhuish et al., 2015). However, the empirical basis of the psychological mechanisms that underlie the protective role of process quality in regular ECEC centers for children exposed to cumulative family risk is still sparse. Given that children spend a large part of their daily lives in ECEC, it is critical to examine its differential as well as long-term effects on children’s development. The present study aimed to address this research gap with a focus on the longitudinal promotive and protective role of different dimensions of process quality (teaching and interaction, provisions for learning, and key professional tasks) in regular center-based ECEC in the association between cumulative early family risks and children’s social–emotional development [i.e., conduct problems (CP), emotional problems (EP), and prosocial behavior (PB)] from early to middle childhood.

### Risk and Resilience

Numerous studies found negative effects of multiple family risk exposure on children’s social–emotional development from early childhood to adulthood (e.g., Burchinal et al., 2006; Pungello et al., 2010; Slopen et al., 2014). Family risk factors are characteristics of family members or of the family as a whole. Examples of family risks are socioeconomic aspects (e.g., poverty, low education of the parents, or single parenthood), interpersonal aspects (e.g., family conflicts, abuse, or maltreatment), critical life events (e.g., death or illness of important persons, frequent moves, and migration), and other risks such as substance abuse or parental mental illness (see Sticca et al., 2020). In particular, the accumulation of family risks is indicated as a major threat to children’s development (e.g., Rutter, 2000; Evans et al., 2013). Early adverse life events and experiences do not occur in isolation in most cases; rather, they are both common and interrelated (Wadman et al., 2020). Many studies have shown that cumulative risk (number of risk factors) is a better predictor of a wide range of developmental outcomes than any single risk factor, indicating that children with high cumulative risk scores have worse social–emotional development (e.g., internalizing and externalizing symptoms) than children with low-risk scores, independent of the specific risks included in the score (e.g., Deater-Deckard et al., 1998; Gutman et al., 2003; Hogye et al., 2022). The occurrence of multiple family risk factors has been, for example, associated with ongoing and early-onset behavior problems (Appleyard et al., 2005; Gach et al., 2018). Additionally, the presence of proximal risk factors is emphasized over distal risk factors (e.g., McLaughlin, 2016).

A body of research has examined the processes by which children’s development is successful and positive despite those multiple psychosocial risks. The term resilience refers to “the capacity of a dynamic system to withstand or recover from significant challenges that threaten its stability, viability, or development” (Masten, 2011, p. 494). The basis for positive development in the face of adversity lies in specific protective factors within the child and the child’s environment. Studying the relation of risk and positive outcomes, a moderator model is emphasized in resilience research (Luthar et al., 2000; Rose et al., 2004). On the one hand, a protective factor buffers the undesirable effects of risks (i.e., moderating or interaction effect). Promotive factors, on the other hand, have a favorable effect regardless of the level of risk (main effect; Sameroff, 2000). Specific factors can be both promotive and protective or just one of each (Masten and Barnes, 2018).

The theoretical embedding of resilience in a process-oriented risk-protection model enables a multifaceted and dynamic view of child development (Rutter, 2012; Masten, 2018). The past decades of empirical research on resilience have shown that resilience is not a fixed inherent personality trait. On the individual side, it can be understood as specific cognitive, motivational, or social–emotional competencies or skills that are age-, domain-, and context-specific, and that enable the child to adapt positively despite a given risk (e.g., Shean, 2015). On the contextual side, the child’s caregivers in the family but also in extrafamilial settings such as ECEC and schools, play an important role, whether in supporting the child in coping with stress and adversities or in developing resilience-enhancing competencies such as, for example, self-efficacy experiences or emotion regulation skills (e.g., Masten and Barnes, 2018). Role models outside the family as well as engagement in a well-functioning school have been found to be potential buffers for children at risk (e.g., Bender and Lösel, 1997; Werner and Smith, 2001). From a developmental psychological perspective, the experience of secure and stable attachments has a generative function. The emotional availability of a significant adult caregiver not only serves as a protective factor affecting the prevention of deviant behavior or mental disorders (Luthar et al., 2000). It also contributes to a mental healthy development, primarily of skills or competencies that can be considered as resilience markers. The supportive environment can provide a substantial foundation for learning new coping mechanisms in the face of stress and thus also lays the foundation for a steeling effect (Rutter, 2012). Daniel and Wassell (2002) summarize three resilience-promoting factors for early childhood that can be seen as important psychological mechanisms: (1) secure attachments, (2) self-esteem, and (3) a sense of self-efficacy. Regular center-based ECEC settings can be a beneficial place of stable and secure relationships, exploration, and encouragement for children at risk.

### Childcare Process Quality and Social–Emotional Development

The importance of high-quality ECEC for children’s development is undisputed (for a review, see, e.g., Anders, 2013; Melhuish et al., 2015). Whereas earlier studies focused mainly on the dosage of ECEC (e.g., duration, intensity, or types of ECEC), quality indicators have also been studied in recent decades. In this context, process quality is highlighted as the most critical dimension of quality in ECEC as it directly affects the child’s development (e.g., Pianta et al., 2005; Kluczniok and Roßbach, 2014). Process quality refers to the proximal processes of children’s experiences in ECEC and includes the social, emotional, physical, and instructional aspects of interactions between caregivers and children as well as among children such as day-to-day interactions, activities, and learning resources (Slot, 2018).

Results on the promotive effects of process quality in ECEC on child development are heterogeneous. Effects depend on the ECEC system and its socio-political context, on children’s age (below vs. above three years), on the outcome of interest, on the measurement of process quality, and, finally, on the duration of the studies. In this context, research findings on children’s social–emotional development are less consistent than on children’s cognitive and language development, specifically for the first years of life (for a review, see, e.g., Anders, 2013; Melhuish et al., 2015). Although most short-, medium-, and long-term effects of high process quality on social–emotional outcomes were positive (e.g., Peisner-Feinberg et al., 2001; Loeb et al., 2004; Sammons et al., 2008, 2014, 2012; Vandell et al., 2010; Votruba-Drzal et al., 2010; Gialamas et al., 2014), zero effects or inconsistent findings were also observed in longitudinal studies (e.g., NICHD Early Child Care Research Network, 2005; Wylie et al., 2006; Stein et al., 2013). Furthermore, the long-term effects were rather weak. It is assumed that this might be due to the measurement of social–emotional outcomes that are mainly based on diverse assessments as well as on subjective reports of parents and teachers and rarely on standardized tests, in contrast to the assessment of cognitive outcomes (Anders, 2013). Moreover, the impact of family characteristics on social–emotional development seemed to be higher than on the cognitive domain, and the development of social–emotional competencies is the result of the interplay of multiple social experiences in childhood (Konrad-Ristau and Burghardt, 2021).

### Childcare Process Quality as Moderator

Several studies to date have examined the protective role of childcare quality for children at various psychosocial risks (e.g., Burchinal et al., 2000; NICHD Early Child Care Research Network, 2002; Dominguez et al., 2011; Sammons et al., 2012; Goelman et al., 2014; Berry et al., 2016; Charrois et al., 2020). However, only a few studies included cumulative/multiple family risks (Burchinal et al., 2006; Hall et al., 2009, 2013; Vandell et al., 2010; Wustmann Seiler et al., 2017) and focused on children’s social–emotional outcomes (NICHD Early Child Care Research Network 2002; Burchinal et al., 2006; Vandell et al., 2010; Hall et al., 2013; Wustmann Seiler et al., 2017). Furthermore, most studies were cross-sectional, with very few examining the protective role of process quality in ECEC longitudinally into middle childhood or adolescence (Burchinal et al., 2006; Vandell et al., 2010). In addition, to date, the available research results are inconsistent. Hall et al. (2013) found protective effects of process quality on preschool-age children’s (age 3–5 years) self-regulation and anti-social behavior for child-related risks but not for family risks. In addition, the protective function of childcare process quality proved to be much stronger for cognitive outcomes than for social–emotional ones. The NICHD Early Child Care Research Network (2002) found buffering effects of childcare process quality on the associations between socio-cultural family risks and young children’s (age 2–3 years) mother-reported prosocial behavior. In contrast, Vandell et al. (2010) found no significant long-term moderation effect of childcare process quality on behavioral outcomes of adolescents (age 15) with early family risks. Burchinal et al. (2006) reported a non-protective effect of childcare process quality on children’s social skills, but a significant protective effect on children’s behavioral problems from kindergarten through third grade among children exposed to multiple risks.

Almost all studies included a global/overall measure of childcare process quality; only very limited studies made a distinction between different dimensions of process quality and their protective function such as quality of interactions or domain-specific learning opportunities. Hall et al. (2009) showed that a high teacher–child interaction quality was found to be protective for child-related risk, while global process quality and the domain-specific quality of the curricular provision were protective factors for family risk regarding children’s cognitive abilities. In a previous short-term study, we reported that high-quality teaching and interaction as well as good provisions for learning—assessed as developmentally appropriate and stimulating spatial and material environments that provide children with all kinds of learning opportunities—mitigated the negative effects of family risks on parent-reported children’s internalizing problems (age 3–5 years; Wustmann Seiler et al., 2017).

The present inconsistent findings on the protective role of process quality in ECEC also fit in with those on compensatory effects for children from disadvantaged backgrounds (e.g., low-socioeconomic/low-income families, families with immigrant or ethnic minority origin, with poor or temporary housing, or from lower educational levels). However, for regular ECEC centers hardly any compensatory effects could be achieved with those children in contrast to more complex and highly specialized intervention programs (e.g., the Head Start Program, the High/Scope Perry Preschool Project, or the Abecedarian Program; for a review, see, e.g., Anders, 2013; Kluczniok, 2017). If compensatory effects were found, they were mostly small. Furthermore, the effects were found to be stronger for cognitive outcomes, for children under 3 years of age, and when combined with associated home visit programs (Melhuish et al., 2015). For example, the international ECCE study (ECCE-Study Group, 1999) found no evidence for a compensatory effect of process quality on children’s social–emotional development from disadvantaged backgrounds, while Sammons et al. (2012) reported compensatory long-term effects of preschool quality on self-regulation and hyperactivity in children aged 14 with a low quality home learning environment.

In conclusion, less is known about the long-term moderating role of process quality in regular center-based ECEC on children’s social–emotional development, notably for children exposed to cumulative family risk. Also, more research is warranted to differentially investigate various dimensions of process quality in ECEC in their buffering effects. This evidence can provide further indications for quality improvement in the regular ECEC systems for policy and practice, as well as for prevention and intervention with children facing multiple risks in early childhood.

### Aims and Hypotheses

**Figure 1:**
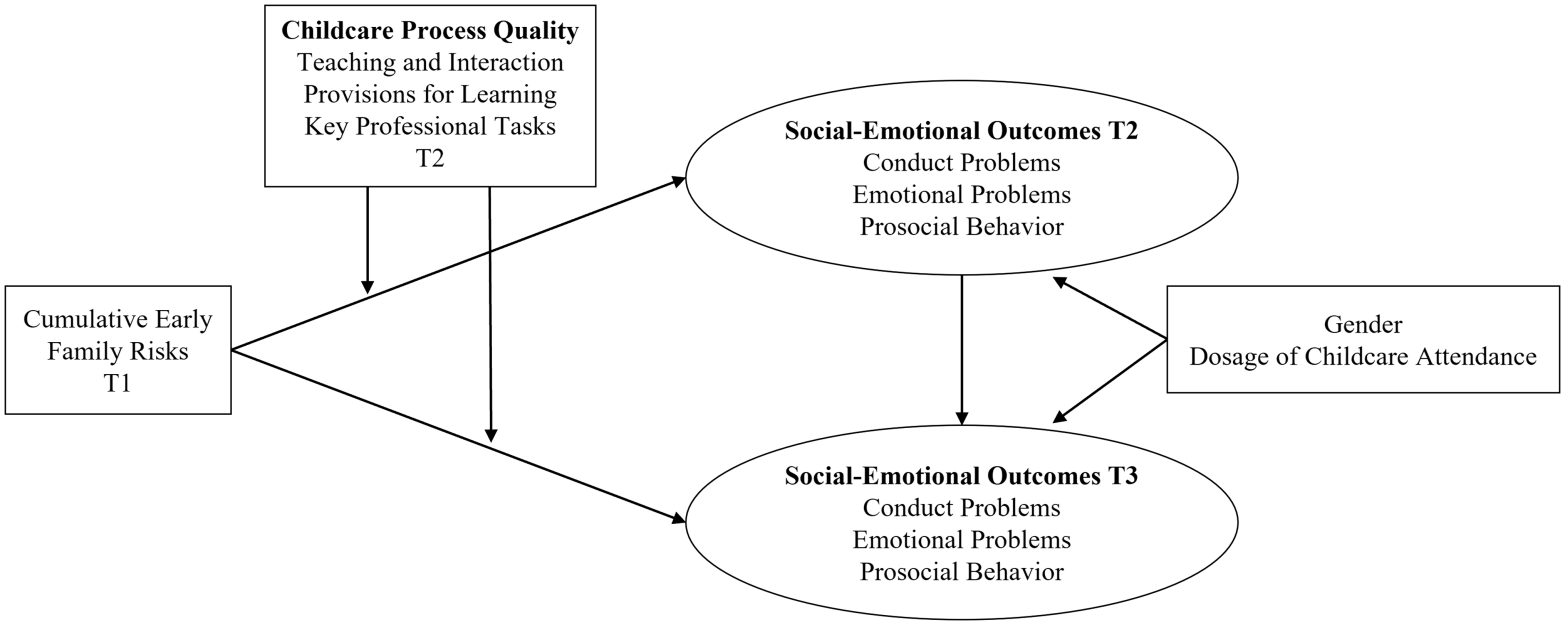
Basic model of the study. Socio-emotional outcomes were calculated using a multi-informant approach (parent and teacher ratings), controlling for gender as well as intensity and duration of childcare attendance. T1, T2, and T3 = waves of assessment.

The present study aimed to extend previous research findings and to investigate long-term promotive and protective effects of process quality in regular center-based ECEC in the context of early family risks and children’s social–emotional development through middle childhood. We examined whether process quality in ECEC (ages 3–5 years) moderates the association between cumulative family risks in early childhood (ages 2–4 years) and social–emotional outcomes (CP, EP, and PB) at a later stage in development (school age; 9–11 years). Figure 1 illustrates a basic model of the present study. Using a multi-informant approach for assessing social–emotional outcomes according to Kraemer et al. (2003) by parent and teacher reports and controlling for the dosage of childcare attendance (i.e., intensity and duration), we hypothesized that (H1a) early family risks (T1) would be positively associated with conduct problems and emotional problems and (H1b) negatively associated with prosocial behavior in middle childhood (T3). We expected that (H2a) childcare process quality (T2) would be negatively linked to conduct problems and emotional problems and (H2b) positively linked to prosocial behavior in preadolescence (T3; long-term promotive effects). Moreover, we hypothesized that childcare process quality buffers the undesirable effects of early family risks (T1) on conduct problems, emotional problems, and prosocial behavior in middle childhood (H3; long-term protective effects).

## Materials and Methods

### Sample and Procedure

The present longitudinal study was part of a larger-scale intervention study (“Promoting Early Learning and Resilience through a Strengthening Learning Dialogue: A Project for Promotion and Professionalization of Early Childhood Education in Swiss Childcare Centers,” 2009–2012) with a follow-up study 6 years later (“Long-Term Effects of Early Family Risk on Children’s Maladjustment and Self-Efficacy: Individual, Familial, and Extra-Familial Protective Processes,” 2016–2019). The overall study involved 25 ECEC centers with 69 groups in Switzerland. It covered three measurement occasions starting in 2009 (T1) and lasting to 2016 (T3). The first measurement (T1) comprised a parent interview and a parent questionnaire on children’s exposure to early family risks. At the second and third measurements (T2/T3), parents and teachers rated children’s social–emotional outcomes. The assessment of childcare process quality was also part of the second measurement (T2).

ECEC centers were all located in cities or agglomeration communities. The size of the centers ranged from one to seven groups. Most of the centers had an average size of 3–4 groups. Since a complete assessment of childcare process quality in all groups (*n* = 75) was not possible for reasons of survey organization and efficiency (half a day per group per assessment), a random sample of groups was drawn. The sampling was based on a calculation principle, which is composed of the number of groups per ECEC center. The underlying assumption is that the scaled number of groups per center represents the quality of all groups in the center: For sizes of 1–2 groups, all groups were included in the sampling; for sizes of 3–4 groups, two groups were randomly selected; for sizes of five or more groups, three groups were randomized. The selection was made using the Research Randomizer. A total of 50 groups were included for the assessment of childcare process quality. Of these, 42 groups enrolled children participating in the present longitudinal study. Other groups consisted only of babies and toddlers under the age of 2 or after-school children aged 7 years and older.

The sample consisted of 293 children between 2 and 4 years (*M*_age_ = 2.81; *SD*_age_ = 0.55; 47.9% female) and their primary caregivers at T1. The number of participating children in each ECEC group varied between 1 and 11 (*M* = 4.25). At T2 in 2010, 239 children (*M*_age_ = 3.76; *SD*_age_ = 0.49; 47.3% female; participation rate 81.6%) and their primary caregivers participated. Finally, at T3, 189 children, now in middle childhood between 9 and 11 years (*M*_age_ = 9.69; *SD*_age_ = 0.48; 48.6% female), took part in the long-term follow-up study 6 years later (79.1% of those participating at T2). Most families were of Swiss origin (89.2%), German-speaking (local language at home, 84.2%), and highly educated (63.1% of mothers had a university degree at T1, 69.7% at T3). The return rates of the questionnaires for rating children’s social–emotional outcomes were 87.0% (T2) resp. 89.4% (T3) for parents and 100% (T2) resp. 90.7% (T3) for teachers.

Dropout rates were relatively small considering that they were approximately 7 years: 103 children left the longitudinal study between T1 and T3. Analyses of missingness revealed that children who participated in both T1 and T2 had comparable scores of early family risks (*ß* = −0.09; *p* = 0.18) to those who participated in T1 only. However, children who participated in all three times of measurements had significantly lower scores of early family risks (*ß* = −0.15; *p* = 0.02) than those who participated in T1 and T2 only. Children who participated in both T2 and T3 had comparable scores of behavioral problems (*ß* = −0.03; *p* = 0.76), emotional problems (*ß* = 0.02; *p* = 0.79), and prosocial behavior (*ß* = 0.01; *p* = 0.95) to children who participated in T2 only. These results point to the presence of a slightly selective dropout, which, however, does not correlate with the socio-emotional outcomes.

### Ethical Procedure

Parents and teachers were informed about the aims and procedures of the study by a written study description and gave their written consent for participation. Children obtained these documents at the third measurement occasion and agreed to participate. Parents, teachers, and children were informed about their right to terminate their participation at any time without stating any reason. In addition, all three were advised that data would be stored on a secure server in anonymized form and used exclusively for research purposes. After each assessment, participating children received a small gift. All procedures were consistent with the Swiss legislation for research with human participants. No ethical approval was needed as confirmed by the Ethics Committee of the Canton of Zurich.

### Study Measures

#### Cumulative Early Family Risks

Given the sensibility of some issues, a combination of a parent interview and a parent questionnaire was applied to assess early family risks at T1. The parent interview was carried out by trained undergraduate students using a standardized procedure that was adapted from various instruments applied in research on family risks (e.g., Rutter and Quinton, 1977; Esser et al., 1989). The aim was to model a cumulative risk score based on both distal and proximal familial risk factors. The following 14 dichotomized risk factors were subsumed to an overall cumulative score of family risks (*M* = 0.08, *SD* = 0.08): single-parent family (10.0%), alcohol and/or drug abuse of father and mother (5.4%), current or previous family violence (3.4%), current or previous chronic partnership disharmony (10.6%), family income below the poverty threshold (12.7%), low maternal education (7.9%), immigrant background of the family (16.8%), serious illness or death of a parent (3.0%), serious illness or death of another family member (2.7%), serious illness or death of a friend (1.0%), serious illness of a sibling (4.1%), self-reported mental health issues of father and/or mother (4.9%), move of the family (25.0%), and current or previous issues with the law of father and/or mother (1.9%). A detailed description of all 14 dichotomized risk factors and the background of this cumulative approach can be found in Sticca et al. (2020). The family risk score was not linked to childcare process quality and children’s gender (see Table 1). Intensity (*r* = 0.28, *p* < 0.001) and duration of childcare attendance (*r* = 0.10, *p* < 0.05) were positively related to family risks.

**Table 1:** Zero-order bivariate correlations among all study variables (*N* = 293).

		M	SD	ICC	1	2	3	4	5	6	7	8	9	10	11	12	13	14	15	16	17	18	19	20	21	22	23	24
1	CPa-T2	1.51	0.47	0.09	1																							
2	CPb-T2	1.32	0.37	0.12	0.46^***^	1																						
3	CPc-T2	1.42	0.41	0.12	0.47^***^	0.54^***^	1																					
4	CPa-T3	1.36	0.40	0.02	0.27^**^	0.20^*^	0.20	1																				
5	CPb-T3	1.23	0.38	0.05	0.01	0.01	0.19^*^	0.31^***^	1																			
6	CPc-T3	1.23	0.36	0.09	0.12	0.11	0.20^*^	0.46^***^	0.51^***^	1																		
7	EPa-T2	1.15	0.30	0.09	0.12	0.11	0.19^*^	0.22^*^	−0.02	0.08	1																	
8	EPb-T2	1.23	0.28	0.04	0.02	0.02	0.12	0.13	−0.08	0.08	0.64^***^	1																
9	EPc-T2	1.50	0.46	0.12	0.04	0.02	0.04	0.10	−0.07	−0.04	0.42^***^	0.47^***^	1															
10	EPa-T3	1.31	0.42	0.06	−0.02	0.03	0.15	0.15	0.17	0.24^**^	0.29^**^	−0.02	0.18^*^	1														
11	EPb-T3	1.17	0.33	0.09	−0.05	0.00	0.14	0.15	0.23^*^	0.24^*^	0.07	−0.04	0.07	0.53^***^	1													
12	EPc-T3	1.36	0.43	0.16	0.12	−0.03	0.13	0.15	0.02	0.19^*^	0.12	0.05	0.21^**^	0.36^***^	0.32^**^	1												
13	PBa-T2	2.53	0.41	0.02	−0.32^***^	−0.34^***^	−0.35^***^	−0.21^*^	−0.16	−0.21^*^	−0.01	0.04	0.09	−0.04	−0.07	0.05	1											
14	PBb-T2	2.61	0.38	0.09	−0.06	−0.25^***^	−0.19^**^	−0.26^*^	−0.29^***^	−0.19^*^	0.00	−0.01	−0.03	0.07	0.04	0.15	0.45^***^	1										
15	PBc-T2	2.38	0.47	0.10	−0.05	−0.24^***^	−0.22^**^	−0.16	−0.10	−0.19^*^	−0.12	−0.08	−0.06	0.04	0.14	0.13	0.34^***^	0.49^***^	1									
16	PBa-T3	2.57	0.40	0.07	−0.05	−0.18^*^	−0.22^*^	−0.48^***^	−0.58^***^	−0.45^***^	0.13	0.17^*^	0.09	−0.05	−0.10	0.05	0.23^*^	0.23^**^	0.15	1								
17	PBb-T3	2.66	0.38	0.11	−0.02	−0.09	−0.03	−0.27^**^	−0.48^***^	−0.47^***^	−0.03	−0.13	−0.04	−0.02	−0.10	0.01	0.15	0.21^**^	0.13	0.44^***^	1							
18	PBc-T3	2.38	0.48	0.10	−0.01	0.00	0.10	−0.33^***^	−0.25^**^	−0.31^***^	−0.11	−0.08	−0.02	−0.07	−0.06	−0.07	0.08	0.24^*^	0.24^***^	0.32^***^	0.54^***^	1						
19	FR-T1	0.08	0.08	0.11	0.11	0.16	0.27^***^	−0.29^**^	0.09	0.25^*^	0.24^**^	0.22^**^	0.17^**^	0.18	0.33^**^	0.12	−0.11	−0.10	−0.10	−0.18^*^	−0.12	−0.18^*^	1					
20	TI-T2	4.99	0.69	−	−0.03	−0.07	−0.14	−0.16	−0.05	−0.17	−0.16	−0.19	0.05	0.06	0.05	−0.08	0.08	0.02	0.12	0.06	0.12	0.02	−0.06	1				
21	PL-T2	2.84	0.59	−	−0.11	−0.19^**^	−0.30^***^	−0.02	−0.02	−0.14	−0.22^**^	−0.22^***^	−0.07	−0.02	−0.07	−0.15^*^	0.09	0.07	0.07	−0.10	0.04	−0.07	−0.12	0.35^*^	1			
22	KP-T2	2.85	0.79	−	−0.01	−0.10	−0.03	0.01	0.14	0.07	−0.17	−0.14	−0.16	−0.03	−0.05	−0.05	0.08	−0.03	0.12	−0.02	−0.01	−0.08	−0.03	0.21	0.49^**^	1		
23	IC-T1	4.32	1.94	0.04	0.14	0.11	0.13	0.13	−0.04	0.25	0.12	0.16	0.02	0.02	0.13	0.15	−0.02	0.11	0.10	−0.01	−0.02	−0.02	0.28^***^	−0.12	−0.17^**^	−0.05	1	
24	DC-T1	3.85	1.73	0.02	−0.06	0.04	0.05	0.05	0.08	−0.11	−0.01	−0.03	0.00	0.01	0.12	−0.01	−0.10	−0.11	−0.04	−0.10	−0.04	0.02	0.10^*^	−0.11	0.01	−0.11	−0.06	1
25	Sex-T1	0.52	0.50	0.00	0.10	0.19^**^	0.19^**^	0.23^**^	0.20^**^	0.15^*^	0.01	0.05	0.04	−0.10	−0.04	0.07	−0.21^***^	−0.31^***^	−0.29^***^	−0.26^***^	−0.33^***^	−0.41^***^	−0.01	0.01	−0.03	−0.02	−0.07	−0.02

CP, conduct problems; EP, emotional problems; PB, prosocial behavior; FR, familial risks; TI, teaching and interaction; PL, provisions for learning; KP, key professional tasks; IC, intensity of childcare; DC, duration of childcare; Sex (1 = male, 0 = female); Letters a,b,c, different items of the various constructs; T1–T2–T3, waves of data assessment; and ICC, intraclass correlation.

* *p* < 0.05;

** *p* < 0.01;

*** *p* < 0.001.

#### Childcare Process Quality

Childcare process quality was assessed through a non-participatory external observation. Observations were performed during one single morning, lasted 4 h each, and were followed up by an interview with the caregiver/teacher in charge. Due to the broad range of children’s age within the majority of ECEC groups (usually 6 months to 5 years; *M* = 3;0, *SD* = 2;1, Min = 0;6, Max = 11;10), two trained observers carried out the observations and rated the childcare process quality using the German versions of the following four instruments: the *Infant/Toddler Environment Rating Scale-Revised* (ITERS-R, 0–3 years; Tietze et al., 2005), the *Early Childhood Environment Rating Scale-Revised* (ECERS-R, 3–6 years; Tietze et al., 2007), the *Early Childhood Environment Rating Scale-Additional items* (ECERS-Z, 3–6 years; Tietze and Roßbach, 2017), and the *Early Childhood Environment Rating Scale-Extension* (ECERS-E, 3–6 years; Roßbach and Tietze, 2018). Observers took part in extensive training by authorized instructors and obtained a certification after completing with an interrater reliability of at least 0.85. Items were rated on a seven-point Likert scale ranging from 1 (inadequate) to 7 (excellent).

The ITERS-R and ECERS-R Rating Scales are categorized into seven subscales: (1) space and furnishings, (2) personal care routines, (3) language and books resp. language-reasoning, (4) activities, (5) interaction, (6) program structure, and (7) parents and staff. Consistent with previous studies (Clifford et al., 2010; Mayer and Beckh, 2017; Mariano et al., 2019), the subscales did not show fully acceptable internal consistency, therefore the following three scales were formed based on other research (e.g., Sakai et al., 2003; Cassidy et al., 2005) as well on exploratory factor analyses in Mplus (version 8.2, Muthén and Muthén, 1998–2017), namely teaching and interaction (nine items from ITERS-R and five items from ECERS-R, e.g., staff-child interactions, interactions among children, supervision of play and learning, discipline), provisions for learning (six items from ITERS-R and 8 items from ECERS-R, e.g., room arrangement for play, using books, art, music and movement, and space for privacy), and key professional tasks (five items from ECERS-Z/ECERS-E, e.g., observation and documentation of the child’s learning and development, planning for children’s individual learning needs, and communication on pedagogical issues within the team and between staff and parents). All model fit indices were satisfactory to good [for ITERS-R *χ*^2^(76) = 85.87, *p* = 0.21, CFI = 0.93, RMSEA = 0.06, SRMR = 0.08, for ECERS-R *χ*^2^(53) = 59.75, *p* = 0.24, CFI = 0.95, RMSEA = 0.06, SRMR = 0.09, and for ECERS-Z/ECERS-E *χ*^2^(5) = 1.47, *p* = 0.92, CFI = 1.00, RMSEA = 0.00, SRMR = 0.05] and factor score determinacy coefficients ranged from 0.86 to 0.97 (Tabachnick and Fidell, 2013). Internal consistency coefficients were found to be sufficient (for teaching and interaction, Cronbach’s Alpha = 0.85, for provisions for learning, Cronbach’s Alpha = 0.64, for key professional tasks, Cronbach’s Alpha = 0.61). More details about the specific instruments, procedures, and scales can be found in Wustmann Seiler et al. (2017, 2019).

#### Social–Emotional Outcomes

Parents and teachers completed the Strengths and Difficulties Questionnaire (SDQ; Goodman, 1997) for a multi-informant approach. The SDQ is widely used and well established to identify the strengths and difficulties of social–emotional outcomes of 3–16-year-olds. The subscales for conduct problems (five items), emotional problems (five items), and prosocial behavior (five items) were used for the present study. All items were assessed using a three-point Likert scale ranging from 1 (not true) to 3 (certainly true). To generate the multi-informant approach, items rated by parents and teachers were averaged. Confirmatory factor analyses were carried out from both T2 and T3. To reduce the complexity of the model and to adhere to the specification of using exactly three indicators for each single latent variable (Little, 2013), we decided to include three items per subscale that met the following criteria: (1) high face validity, (2) satisfactory reliability, and (3) no problematic pattern of error correlations (see also Sticca et al., 2020). The model of conduct problems contained the items *“often has temper tantrums or hot tempers,” “often fights with other children or bullies them,”* and *“generally obedient, usually does what adults request (recoded).”* The items *“many worries, often seems worried,” “often unhappy, down-hearted or tearful,”* and *“nervous or clingy in new situations, easily loses confidence”* were selected for the model of emotional problems. The model of prosocial behavior included the items *“considerate of other people’s feelings,” “helpful if someone is hurt, upset or feeling ill,”* and *“often volunteers to help others (parents, teachers, other children).”* The resulting models with two latent variables (T2 and T3, with three indicators each) and only longitudinal correlations fitted the data very well [conduct problems, *χ*^2^(5) = 6.93, *p* = 0.23, CFI = 0.99, RMSEA = 0.04, SRMR = 0.04; emotional problems, *χ*^2^(5) = 6.65, *p* = 0.25, CFI = 0.99, RMSEA = 0.04, SRMR = 0.04; and prosocial behavior, *χ*^2^(5) = 1.30, *p* = 0.94, CFI = 1.00, RMSEA = 0.00, SRMR = 0.02]. McDonald’s omega reliability values were found to be adequate (conduct problems 0.74 at T2 and 0.71 at T3; emotional problems 0.76 at T2 and 0.65 at T3; prosocial behavior 0.70 at T2 and 0.72 at T3). Descriptive statistics are reported in Table 1.

Measurement invariance was tested by comparing a configural model with unconstrained item loadings and intercepts to a metric invariance model with item loadings constrained to equality, and finally by comparing the metric model to a scalar invariance model with item intercepts constrained to equality (Cheung and Rensvold, 2002). Measurement results are reported in Table 2. Results showed that for all latent variables the metric invariance constraints did not lead to a deterioration of model fit. In contrast, the scalar invariance constraints led to a strong model fit deterioration for emotional problems. This finding indicates that the way the various items represent the latent construct of emotional problems was stable over time, however, the differences between the mean values of the items and the mean values of the latent construct were not stable (see also Sticca et al., 2020). This measurement invariance pattern enables comparison of variances and covariances, but not that of means and intercepts (Cheung and Rensvold, 2002). Because the present study did not focus on a comparison of means over time, we applied only the constraints of metric invariance in all longitudinal models.

**Table 2:** Model fit comparison of the three social-emotional outcomes outcomes for the examination of measurement invariance.

	*χ* ^2^	*df*	*p*	CFI	RMSEA	SRMR	∆*χ*^2^	∆*df*	*p*
**Conduct problems**									
Configural	6.93	5	0.226	0.99	0.04	0.04			
Metric	7.22	7	0.406	1.00	0.01	0.05	0.29	2	0.732
Scalar	12.08	9	0.209	0.98	0.04	0.05	4.86	2	0.066
**Emotional problems**									
Configural	6.65	5	0.248	0.99	0.04	0.04			
Metric	11.12	7	0.134	0.98	0.05	0.06	4.47	2	0.110
Scalar	51.21	9	0.000	0.79	0.15	0.09	40.09	2	0.000
**Prosocial behavior**									
Configural	1.30	5	0.936	1.00	0.00	0.02			
Metric	1.85	7	0.968	1.00	0.00	0.04	0.55	2	0.756
Scalar	2.63	9	0.977	1.00	0.00	0.05	0.78	2	0.650

*χ*^2^, chi-square; *df*, degrees of freedom; *p*, probability of type I error; CFI, comparative fit index; RMSEA, root mean square error of approximation; SRMR, standardized root mean square residual; and ∆, difference value.

#### Dosage of Childcare Attendance

In the context of the parent interview, parents reported on the intensity of childcare attendance in steps of half-days ranging from 1 (1 day) to 9 (5 days). A total of 58.9% of the children spent less than 3 days per week at the ECEC center, while the remaining 51.1% spent 3 or more days. In addition, parents reported since when their child attended the current ECEC center. The frequencies for the respective ages of entrance were 1.7% for under 3 months, 24.3% for 3–6 months, 24.0% for 7–12 months, 30.5% for 1–2 years, and 19.5% for 2–4 years. Intensity of childcare attendance was negatively related to provisions for learning (*r* = −0.17, *p* < 0.05).

### Analysis Strategy

**Figure 2:**
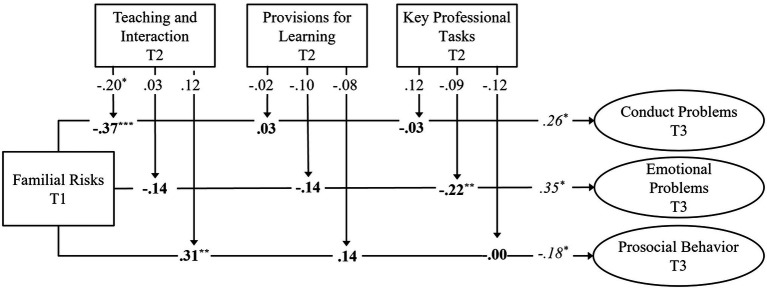
Simplified example of the statistical model for the examination of long-term moderating effects. Bold: Standardized interaction effects (protective effects); *Italics*: Standardized main effects of early family risks; Plain: Standardized main effects of childcare process quality (promotive effects). The covariates (gender, intensity and duration of childcare attendance, and socio-emotional outcomes at T2) are not shown for simplicity. Results come from nine different structural equation models. T1, T2, and T3 = waves of assessment. ^*^p < 0.05; ^**^p < 0.01; and ^***^p < 0.001.

In the present study, we adapted the approach of defining resilience as a process in which the effect of cumulative risks on a given outcome is buffered by a specific protective factor (acting as a moderator, e.g., Luthar et al., 2000; Rose et al., 2004). Once the preliminary confirmatory analyses were completed, a total of nine structural equation models were constructed using Mplus (version 8.2., Muthén and Muthén, 1998–2017), one for each possible combination of the three moderators of childcare process quality (teaching and interaction, provisions for learning, and key professional tasks) and the three dependent variables of social–emotional outcomes (conduct problems, emotional problems, and prosocial behavior; see Figure 1). All nine models had the same structural setup. A sample model for teaching and interaction as a moderator and conduct problems as an outcome variable will be described in detail in the following. In a first step, conduct problems at T2 (CP2) and T3 (CP3) were modeled as a latent outcome variable with the same three indicators. Furthermore, conduct problems at T2 (CP2) were modeled as a latent predictor of conduct problems at T3 (CP3). The effect coding method was used (Little, 2013). Autocorrelations between the same items over time were allowed for all indicators. In a second step, family risks (T1) and teaching and interaction (T2) were added to the model as grand-mean centered manifest variables and modeled as a predictor of conduct problems at both T2 (i.e., short-term effect) and T3 (i.e., long-term effect) together with their manifest interaction term (i.e., protective effect). In a third step, gender, intensity, and duration of childcare attendance were entered into the model as manifest predictors of both CP2 and CP3. All exogenous predictors were allowed to correlate with each other. The evaluation of model fit was based on conventional goodness-of-fit criteria (e.g., Kline, 2005; Hooper et al., 2008). All models fitted the data well (see Table 3). A simplified graphical representation of the statistical model described above can be found in Figure 2.

**Table 3:** Model fit indices of the structural equation models.

	*χ* ^2^	*df*	*p*	CFI	RMSEA	SRMR
**Conduct problems**						
Model for teaching and interaction	53.00	31	0.008	0.91	0.05	0.06
Model for provisions for learning	45.37	31	0.046	0.94	0.04	0.05
Model for key professional tasks	48.00	31	0.026	0.93	0.04	0.05
**Emotional problems**						
Model for teaching and interaction	34.18	31	0.318	0.99	0.02	0.05
Model for provisions for learning	37.62	31	0.192	0.97	0.03	0.05
Model for key professional tasks	28.56	31	0.592	1.00	0.00	0.05
**Prosocial behavior**						
Model for teaching and interaction	17.43	31	0.976	1.00	0.00	0.04
Model for provisions for learning	22.52	31	0.798	1.00	0.00	0.03
Model for key professional tasks	20.15	31	0.933	1.00	0.00	0.04

*χ*^2^, chi-square; *df*, degrees of freedom; *p*, probability of type I error; CFI, comparative fit index; RMSEA, root mean square error of approximation; and SRMR, standardized root mean square residual. Models included covariates of gender, intensity and duration of childcare, and socio-emotional outcomes at T2.

The multilevel data structure of children nested in ECEC groups was considered using the Huber-White sandwich estimator (Freedman, 2006). Intra-class correlations (ICC) for the study variables varied between 0.02 and 0.12 (see Table 1). Because the average number of children in each ECEC group was low (74% of the groups included 1–4 participating children), it indicated that the use of a multilevel model was not appropriate. Finally, missing data were addressed using the Full Information Maximum Likelihood method (FIML).

## Results

Results of the structural equation models that were constructed for each one of the three social–emotional outcomes (conduct problems, emotional problems, and prosocial behavior), and the three moderators of childcare process quality (teaching and interaction, provisions for learning, and key professional tasks) are reported below. Results pertaining to the longitudinal role of early family risks will be outlined first, followed by the prediction of the outcomes in middle childhood at T3 (long-term perspective). Results on the long-term promotive role of childcare process quality regardless of the early family risks will also be reported. However, results on the prediction of the outcomes in early childhood at T2 (short-term perspective) are not specifically described. Nevertheless, they can be found in Tables 4–6. Thus, the results regarding the prediction of conduct problems and emotional problems at T2 (short-term promotive and protective effects) are also not entirely consistent with those reported in Wustmann Seiler et al. (2017). Certain deviations in the magnitude and significance of the effects can be attributed to (a) the application of the multi-informant approach (parents and teacher rating), (b) the use of the effect coding method, (c) a larger sample size, and (d) a different pool of variables modeled for the outcomes (three indicators per outcome). Given the inclusion of the three outcomes at T2 as predictors of the outcomes at T3 and the non-modeled scalar invariance, the effects on the various outcomes at T3 can be interpreted as a prediction that controlled for T2 (see Figure 1).

**Table 4:** Standardized regression coefficients of the model for conduct problems (*N* = 293).

	Teaching and interaction		Provision for learning		Key professional tasks	
	CP T2	CP T3	CP T2	CP T3	CP T2	CP T3
CP T2		0.21		0.19		0.20
Family risks	0.24^**^	0.21^*^	0.23^*^	0.26^*^	0.23^**^	0.25^†^
CPQ	−0.09	−0.20^*^	−0.27^***^	−0.02	0.05	0.12
Family risks * CPQ	−0.04	−0.37^***^	0.04	0.03	0.27^**^	−0.03
Intensity of care	0.11	0.18	0.09	0.15	0.13	0.12
Duration of care	0.00	−0.09	0.02	−0.05	0.05	−0.04
Sex (male)	0.23^**^	0.16^*^	0.23^**^	0.21^*^	0.24^***^	0.23^**^

CP, conduct problems; T2, second wave of assessment; T3, third wave of assessment; and CPQ, childcare process quality.

† *p* < 0.10;

* *p* < 0.05;

** *p* < 0.01;

*** *p* < 0.001.

**Table 5:** Standardized regression coefficients of the model for emotional problems (*N* = 293).

	Teaching and interaction		Provision for learning		Key professional tasks	
	EP T2	EP T3	EP T2	EP T3	EP T2	EP T3
EP T2		−0.05		−0.10		−0.03
Family risks	0.24^**^	0.35^*^	0.22^*^	0.32^*^	0.25^**^	0.37^**^
CPQ	−0.19^*^	0.03	−0.22^**^	−0.10	−0.16	−0.09
Family risks * CPQ	−0.09	−0.14	−0.15	−0.14	0.10	−0.22^**^
Intensity of care	0.06	0.04	0.05	0.03	0.09	0.03
Duration of care	−0.06	0.05	−0.03	0.03	−0.04	0.00
Sex (male)	0.05	−0.03	0.06	−0.03	0.06	−0.02

EP, emotional problems; T2, second wave of assessment; T3, third wave of assessment; and CPQ, childcare process quality.

* *p* < 0.05;

** *p* < 0.01;

^***^p < 0.001.

**Table 6:** Standardized regression coefficients of the model for prosocial behavior (*N* = 293).

	Teaching and interaction		Provision for learning		Key professional tasks	
	PB T2	PB T3	PB T2	PB T3	PB T2	PB T3
PB T2		0.17		0.23^†^		0.21
Family risks	−0.16^*^	−0.19^*^	−0.16^†^	−0.15	−0.16^†^	−0.17^†^
CPQ	0.10	0.12	0.09	−0.08	0.06	−0.12
Family risks * CPQ	0.06	0.31^**^	−0.05	0.14	−0.04	0.00
Intensity of care	0.14^†^	−0.05	0.13	−0.04	0.12	−0.03
Duration of care	−0.09	−0.03	−0.10	−0.02	−0.10	−0.04
Sex (male)	−0.41^***^	−0.39^***^	−0.41^***^	−0.40^***^	−0.42^***^	−0.40^***^

PB, prosocial behavior; T2, second wave of assessment; T3, third wave of assessment; and CPQ, childcare process quality.

† *p* < 0.10;

* *p* < 0.05;

** *p* < 0.01;

*** *p* < 0.001.

### Longitudinal Role of Cumulative Early Family Risks

The results on the role of cumulative early family risks are presented in Figure 2. As for the prediction of conduct problems and emotional problems at T3, early family risks (T1) were found to have a positive, small-to-medium, and significant long-term effect. Regarding the prediction of prosocial behavior at T3, early family risks had a negative, small, and significant long-term effect. Thus, children with higher scores of early family risks tended to have more conduct problems, more emotional problems, and less prosocial behavior in middle childhood.

### Results on the Prediction of Conduct Problems

Standardized results of the three models for the prediction of CP are reported in Table 4. Regarding the long-term prediction of CP at T3, teaching and interaction had a negative, small, and significant main effect, while effects of provisions for learning and key professional key professional tasks were found to be negligible. In other words, higher scores of teaching and interaction were linked to lower scores of CP in middle childhood (i.e., long-term promotive effect). Turning to the moderating role of childcare process quality, teaching and interaction had a negative, medium, and significant moderating effect (i.e., long-term protective effect), while the effects of provisions for learning and key professional tasks were virtually zero. These results suggest that high-quality teaching and interaction in ECEC were found to have a long-term link to conduct problems in middle childhood and to moderate the effects of early family risks on school-age children’s conduct problems. Regarding the covariates, intensity of childcare attendance had a positive, small, non-significant effect, while duration of childcare attendance had no noticeable effects on CP at T3. Boys were found to have slightly higher levels of CP at T3 as compared to girls.

### Results on the Prediction of Emotional Problems

Standardized results of the three models for the prediction of EP are reported in Table 5. Results regarding the long-term prediction of EP at T3 showed that all three dimensions of childcare process quality had a negligible effect on emotional problems in middle childhood (i.e., long-term promotive effect). Key professional tasks were found to have a negative, small-to-medium, significant buffering effect, while the interplays between family risks and teaching and interaction and provisions for learning were found to be small and non-significant. These findings suggest that the implementation of key professional tasks in ECEC has a long-term protective effect for children exposed to early family risk on emotional problems in middle childhood. Turning to the covariates, no effects of meaningful magnitude were found.

### Results on the Prediction of Prosocial Behavior

Standardized results of the three models for the prediction of PB are reported in Table 6. Results regarding the long-term prediction of PB at T3 showed that no meaningful promotive effects of childcare process quality were observed. All effects were small and non-significant. The moderating effect between early family risks and teaching and interaction was found to be positive, medium, and significant, while the interactions between family risks and provisions for learning and key professional tasks were found to be small to virtually zero and non-significant. These results indicate that high-quality teaching and interaction in ECEC have a long-term buffering effect on prosocial behavior in school-aged children at risk. Regarding covariates, no meaningful effects were identified for intensity and duration of childcare attendance, while boys had remarkably and significantly lower scores of prosocial behavior in middle childhood than girls.

## Discussion

To date, only limited evidence is available on the long-term protective effects of childcare process quality in regular center-based ECEC on the social–emotional development of children exposed to cumulative family risks. The aim of the present study was to investigate the longitudinal promotive and protective role of process quality in ECEC in the context of early family risks and children’s social–emotional outcomes from early to middle childhood. Specifically, we examined three different dimensions of childcare process quality (namely teaching and interaction, provisions for learning, and key professional tasks), analyzing long-term promotive and protective effects, and considering children’s social–emotional outcomes (conduct problems, emotional problems, and prosocial behavior) from a multi-informant approach. Results will be discussed starting from the role of cumulative early family risks for children’s social–emotional development, followed by the childcare process quality as a promotive and protective factor in the long-term perspective.

### Effects of Early Family Risks

As previously reported in Wustmann Seiler et al. (2017), family risks in early childhood (2–4 years) were linked to more conduct problems, more emotional problems, and less prosocial behavior in the short-term perspective of early childhood (3–5-years-old). Additionally, the present longitudinal extension suggests that these effects remained present in the long term and were persistent until middle childhood (9–11-years-old). These findings confirmed hypotheses 1a and 1b and are consistent with a variety of other studies that have empirically documented the negative effects of early family risks on children’s socio-emotional development (e.g., Appleyard et al., 2005; Burchinal et al., 2006; Pungello et al., 2010; Slopen et al., 2014; Gach et al., 2018). As formerly noted by Sticca et al. (2020), the prevalence of the 14 early family risk factors varied from quite low rates (e.g., serious illness or death of a friend with a prevalence rate of 1%) to quite high rates (e.g., family relocation with 25%). Even though the present study did not involve a high-risk sample, it nevertheless shows the unfavorable effects of exposure to early cumulative risks. We would expect the negative long-term effects of early multiple family risks to be even higher in a high-risk group as opposed to the community-based sample at hand.

### Promotive Effects of Childcare Process Quality

Higher scores of teaching and interaction in ECEC were linked to lower scores of conduct problems in middle childhood. This finding is in line with other longitudinal studies demonstrating positive long-term effects of high process quality in ECEC on children’s social–emotional development (e.g., ECCE-Study Group, 1999; Gialamas et al., 2014; Sammons et al., 2008, 2014, 2012; Gialamas et al., 2014). Hence, hypothesis 2a is partly confirmed. However, for the other two outcome variables (emotional problems, prosocial behavior), the hypotheses must be rejected (2a, 2b), which is in accordance, for example, with the study by Stein et al. (2013). Yet, the results are still encouraging and enrich the discussion about the long-term beneficial role of ECEC for social–emotional development in children. The quality of interactions plays an important role in ECEC. Several studies have also shown that the quality of interactions both between children and between adults and children referred to as Sustained Shared Thinking (SST), has a positive effect on children’s playful learning (e.g., Siraj-Blatchford et al., 2002). Nonetheless, the rather low level of many ECEC’s quality can be seen as a public health issue. Consistent with other studies, the process quality of Swiss ECEC in the present study was also low to mediocre. The scores were especially low in provisions for learning and key professional tasks, which demonstrates a great need for quality improvement in training, policy, and practice.

### Protective Effects of Childcare Process Quality

The present study demonstrated long-term protective effects of process quality in regular center-based ECEC for children exposed to early family risks. Hypothesis 3 was confirmed. The results fit in with those of Burchinal et al. (2006). High-quality teaching and interaction in ECEC were found to have a long-term buffering effect on conduct problems and prosocial behavior in school-aged children at risk. For caregivers’ professional behavior, a long-term protective effect was found on emotional problems through middle childhood. However, provisions for learning did not reveal any significant long-term promotive or protective effect. Even the long-term effects were small to moderate, the results are beneficial and may strengthen the significance of the protective role of ECEC. Luthar et al. (2006) argued that protective factors should meet four criteria for prevention and intervention. They should be salient in a particular life context, i.e., relevant to a large proportion of the population. They should be malleable, i.e., able to be changed through intervention. They should have continuity, i.e., be effective over time. Finally, they should be generative, i.e., they should catalyze further protective processes. All these criteria can apply to ECEC contexts. However, if ECEC settings are of low process quality, they can become a “double risk” for children at risk. In this case, a low process quality in ECEC can constitute a further threat to a positive social–emotional development of children exposed to family risks (Watamura et al., 2011). This aspect is important to keep in mind and makes the discussion about quality improvement indispensable, particularly considering low to mediocre average levels of process quality in ECEC.

Longitudinal findings of the dosage of ECEC on children’s social–emotional development were found to be non-significant and zero to low in the present study. These results were referred to both duration and intensity of childcare attendance and were in line with other longitudinal studies (for a review, see Anders, 2013; Huston et al., 2015). Positive, small, but non-significant long-term effects were found only for intensity of childcare and conduct problems. At this point, for example, the results agree with another recent Swiss study by Averdijk et al. (2022). Furthermore, it is relevant to consider that most children in Swiss ECEC centers are cared for only part-time and rarely full-time.

### Strengths and Limitations

To the best of our knowledge, this was the first study to examine the protective role of different dimensions of process quality in ECEC in the context of cumulative early family risks and children’s social–emotional development from early to middle childhood. We applied a multi-informant approach by including parent and teacher reports and we included 14 family risk factors to a cumulative risk index. Furthermore, we analyzed promotive and protective effects of different dimensions of process quality in ECEC simultaneously with the use of sophisticated statistical methods. However, the present study faced several limitations. First, the sample size was rather small and there was a slight selective dropout over the 7 years. In addition, process quality in ECEC could not be assessed in all groups of the 25 centers but it was still a random sampling. Second, we could not control for the quality of the home learning environment in early childhood as well as for the effects of primary school education and after-school care in middle childhood. Third, we did not account for current family risks in school age, other relevant child characteristics (e.g., temperament), or other developmental outcomes of interest (e.g., multidimensional self-concept). Self-assessment of social–emotional competencies at school age might also be of relevance. Lastly, because of the lack of scalar invariance in emotional problems, we could not compare the means of the two measurement occasions in all scales (see Sticca et al., 2020).

### Implications for Practice

As described above, a trusting and appreciative relationship to significant adult caregivers outside the family is an essential foundation for developing resilience in children exposed to multiple risks (Werner and Smith, 2001). Adult caregivers in ECEC can act as significant social role models who support adequate coping behaviors and provide responsiveness and age-appropriate stimulation for experiencing one’s own efficacy (Wustmann, 2011). Through systematic observation and documentation of children’s learning and development, the caregivers can be aware of the child’s needs and interests and respond to them in a supportive stimulating manner. They can encourage the child to perceive his or her own abilities, strengths, and milestones. Together, they can discuss what the child might need for the next developmental step and how she or he can be further supported at home and in the ECEC center as part of an “educational partnership” with the families. In addition, adult caregivers in ECEC can be an essential resource for stressed parents. They can be role models of how to interact and deal with children in ways that are beneficial to their development, and they can use their observations and documentations to discuss the child’s competencies with its main caregivers. A very good example of such practice in terms of key professional tasks is the widely used “learning stories” approach in ECEC settings (Carr and Lee, 2012). The knowledge of one’s own strengths, the experience of having achieved something special and being responsible for it, as well as the experience of becoming controlling one’s own behavior are fundamentals for facing challenges and building stable social relationships in life (Werner and Smith, 2001). The experience “I am effective,” “I am valuable,” and “I am respected” gives the child self-confidence and an awareness of his/her own abilities, particularly in times of stress and desolate circumstances. We should be much more aware of this preventive potential in everyday ECEC contexts. Although the empirical evidence for the protective role of ECEC quality is still rather limited, we should keep in mind the individual support of at-risk children in daily interactions in ECEC settings. Here, children can accumulate significant experiences of social relations in socially mixed groups with peers and adult caregivers outside the family (Melhuish et al., 2015). However, process quality in ECEC settings is critical. For policy and practice, this means that we need to keep a constant eye on improving process quality in ECEC.

## Conclusion

The present study highlights the great importance of ECEC for prevention and intervention. The study indicated that the protective effect of process quality in center-based ECEC is still evident in middle childhood. High-quality teaching and interaction with adult caregivers and other children, as well as caregivers’ professional behavior served as long-term protective factors for fewer conduct problems, fewer emotional problems, and more prosocial behavior in school-age children in the face of early family risks. In addition, high-quality teaching and interaction were found to be beneficial for all children in the long-term in terms of fewer conduct problems in middle childhood.

## Data Availability Statement

The raw data supporting the conclusions of this article will be made available by the authors, without undue reservation.

## Author Contributions

CW designed and managed the study (as co-applicant), analyzed and interpreted the data, and wrote the first draft of the manuscript with valuable feedback from all authors. FS was responsible for the entire data management and data analyses strategy for the project and helped to analyze the data. OG-H helped in recruiting the sample and organized data collection. HS was responsible for funding and administering the study (main applicant). CW, FS, OG-H, and HS critically reviewed and revised the manuscript. All authors contributed to the article and approved the submitted version.

## Funding

This study was supported by the Swiss National Science Foundation (Grant Nos. 100019_166003 and 100014_124949) and the Jacobs Foundation.

## Conflict of Interest

The authors declare that the research was conducted in the absence of any commercial or financial relationships that could be construed as a potential conflict of interest.

## Publisher’s Note

All claims expressed in this article are solely those of the authors and do not necessarily represent those of their affiliated organizations, or those of the publisher, the editors and the reviewers. Any product that may be evaluated in this article, or claim that may be made by its manufacturer, is not guaranteed or endorsed by the publisher.
